# Effectiveness of peer counselling in addressing psychological distress and exposure to violence among sexual and gender minorities in Nepal: a study protocol for a randomized controlled trial

**DOI:** 10.1186/s13063-026-09843-9

**Published:** 2026-06-16

**Authors:** Keshab Deuba, Rachana Shrestha, Reena Koju, Manisha Dhakal, James Underhill, Per Einar Binder, Ingvild Fossgard Sandøy, the SAATHI study group, Sanu Shrestha, Runa Tandukar, Sulabh Ghimire, Achyut Lamichane, Sanjay Sharma, Pinky Gurung, Jayanti Dhungana

**Affiliations:** 1 Public Health and Environment Research Centre (PERC), Lalitpur, Nepal; 2 Global Health Epidemiology Group, Centre for International Health (CIH), Department of Global Public Health and Primary Care, University of Bergen, Bergen, Norway; 3 Bergen Centre for Ethics and Priority Setting in Health (BCEPS), Department of Global Public Health and Primary Care, University of Bergen, Bergen, Norway; 4 Blue Diamond Society (BDS), Kathmandu, Nepal; 5 Independent Researcher, Kingsbridge, UK; 6 Department of Clinical Psychology, University of Bergen, Bergen, Norway

**Keywords:** Mental health, Violence prevention, Peer-led intervention, LGBTIQA +, Community-based participatory research, Randomized controlled trial, Nepal

## Abstract

**Background:**

The burden of social stressors (discrimination, prejudice, violence, and stigmatization) is disproportionately high among LGBTIQA + (Lesbian, Gay, Bisexual, Transgender, Intersex, Queer and Asexual) individuals. These stressors negatively influence the overall well-being of this population which in turn affects their health-seeking behaviours, including the disclosure of psychosocial concerns to health workers. Peer-led mental health interventions have therefore been identified as a potentially suitable and acceptable approach for this population. This protocol presents the SAATHI (Support, Advocacy, and Awareness for Trauma, Healing and Inclusion) study. The study will assess the feasibility, acceptability, and effectiveness of peer counselling, safety planning, and group-based sessions in reducing exposure to violence and psychological distress among LGBTIQA + individuals in Nepal.

**Methods:**

We will conduct a three-arm randomized controlled trial with a 1:1:1 allocation ratio. At least 1062 LGBTIQA + participants will be enrolled. Eligibility criteria include age 18–55 years, a history of violence in the past 12 months, and a score of ≥ 3 on the General Health Questionnaire, indicating psychological distress. Participants in the intervention arms will receive six 90-min peer-led counselling sessions and one of the arms will receive monthly peer-led group sessions for shared reflection and support. Follow-up assessments will occur at 7 weeks, 18 weeks, and 12 months post-baseline. A qualitative process evaluation will be conducted of the intervention delivery. The primary outcomes are psychological distress and exposure to violence. The secondary outcomes include post-traumatic stress disorder, participant-reported psychological outcomes, daily functioning, self-esteem, self-compassion, perceived social support, use of personal safety strategies, and satisfaction with community resources. We will analyse quantitative data using intention-to-treat principles. For the qualitative data, our approach will be reflexive thematic analysis, which is grounded in a critical realist/contextualist ontology.

**Discussion:**

This study ultimately aims to reduce health disparities and improve the health and well-being of LGBTIQA + individuals. If effective, the SAATHI intervention has potential to be sustainable through empowered peer-led networks and scalable in low- and middle-income countries due to its low-cost, community-implemented design.

**Trial registration:**

ClinicalTrials.gov NCT06979193. Registered on 20 May 2025, https://clinicaltrials.gov/study/NCT06979193

**Supplementary Information:**

The online version contains supplementary material available at 10.1186/s13063-026-09843-9.

## Background

Mental disorders remain among the top ten leading causes of health loss worldwide with almost 14% of the global population being affected in 2021 [1]. The Global Burden of Disease study, conducted across 204 countries and territories, found that the burden of mental disorders was stable between 1990 and 2019 [2] with anxiety and depressive disorders being the most common across age groups and geographic locations [1].

The prevalence of mental disorders (depression, anxiety, suicide attempts, and suicidal ideation) is much higher among lesbian, gay, bisexual, transgender, intersex, queer, asexual, and other sexual and gender diverse (LGBTIQA +) individuals than in the general population [3–5]. This disparity stems from the widespread hostility, stigma, and systemic discrimination which these marginalized populations regularly face in society and the labour market. In addition, they commonly experience financial challenges due to lack of employment, and rejection, harassment, violence, and lack of support from family and community members [3, 6–13]. The latter not only increases the risk of psychological and physical injuries but also reinforces social isolation. The chronic tension resulting from all these factors is referred to as minority stress [14]. In many resource-limited settings, the added burden of structural inequality (such as the criminalization of same-sex relationships, lack of legal gender recognition, and police harassment or arbitrary arrest), violence, and limited access to health services act as a syndemic to further exacerbate health risks among marginalized and vulnerable populations like LGBTIQA + [15].

Despite suffering a high burden of mental disorders, LGBTIQA + tend to use health services less than the majority population in many countries due to fears of discrimination from health professionals and prior experiences of disrespectful treatment [6, 16]. This may reflect both prejudice, stigma, and a lack of awareness and training among health workers regarding the specific health needs of sexual and gender minorities [6, 16]. The lack of appropriate care has broad-reaching impacts on the psychological well-being, physical health, and productivity of these vulnerable citizens [16, 17]. Untreated depression and anxiety significantly weaken daily functioning and increase the risk of suicidal ideation or behaviours in this population [18]. Thus, lack of inclusive mental health services exacerbates the existing health disparities.

Abuse against LGBTIQA + individuals is often occurs with impunity, also in settings where same-sex relationships are legal. This is due to either weak legal protection of LGBTIQA + individuals or deeply rooted homophobic cultural norms. Due to expectations that they will encounter rejection and discrimination when interacting with majority groups, many LGBTIQA + end up being constantly on the alert, a large proportion hide their identity and/or subject themselves to self-stigma. This negatively affects their self-acceptance and may exacerbate the psychological distress they experience [16].

Nepal is a good example of a conservative society where LGBTIQA + face a high risk of minority stress, violence, abuse, discrimination, and marginalization [19–23]. Despite same-sex sexual activity being legal since 2007, violence perpetrated by partners, clients, family, and police is common, much of which is under-reported [21, 24, 25]. While the constitution of Nepal secures the right to employment for sexual and gender minorities in state structures on the basis of the principle of inclusion, they are still deprived of many employment opportunities [26]. As a consequence, a substantial proportion of them (70%) engage in sex work [27]. A 2021 study conducted among transwomen in the Kathmandu valley reported that 71% of participants felt unaccepted by the Nepalese society, and around 90% concealed their gender identity from their family because of stigma and discrimination [26].

A study conducted among sexual and gender minorities across 15 districts in Nepal found that 61% experienced symptoms of depression in the past week, and 47% reported suicidal ideation in the last 12 months [21]. The prevalence of anxiety and depression among adults in the general population is estimated to be 17% and 12%, respectively [28]. Despite the high burden of disease attributable to mental disorders, mental health remains a low priority in Nepal with minimal government budget allocations (mental health constitutes only 0.2% of the total expenditure on health [29]) and the health workforce consists of only 0.68 psychiatrists and 0.17 psychiatric nurses per 100,000 population [30]. The available services are also not sensitive to the unique needs of sexual and gender minorities, despite their high risk of mental health problems.

To meet the mental health needs of LGBTIQA + people in low- and middle-income countries (LMICs) and thus reduce health inequalities, interventions are needed that are culturally relevant, based on respect for this marginalized group, low cost, and do not require highly trained professionals for delivery considering the few mental health specialists available. In addition, harmful social influences should ideally be reduced, such as exposure to violence, marginalization, and social isolation. However, effective and culturally relevant solutions for LGBTIQA + communities in LMICs are still limited, and their impact is uncertain. Fortunately, a growing but limited number of studies indicate that psychological interventions delivered by non-specialists can partly fill gaps in mental health services [31].

One promising intervention, developed by the World Health Organization (WHO), is Problem Management Plus (PM +), which can be administered by non-specialist health care workers or trained peer or lay providers in a community setting [32, 33]. This intervention is based on established cognitive behavioural therapy and problem-solving techniques. It is designed as an individual or group-based psychological help for adults impaired by distress in communities exposed to adversity. PM + has been evaluated in randomized controlled trials and found to be effective (showing overall moderate to large effect sizes) in reducing symptoms of common mental health disorders, including psychological distress [31]. These studies have focused on populations such as women experiencing gender-based violence, refugees, internally displaced persons, and members of the general population affected by humanitarian crises, including armed conflict, earthquakes, or chronic health conditions like HIV or multiple myeloma [31]. In some of these studies, PM + has been delivered by peers or lay providers [31]. PM + has also demonstrated scalability and adaptability in several LMICs due to its format, reliance on non-specialist or peer providers, and modular design, which permits adaption to varied cultural and contextual needs [31, 34].

Considering that many LGBTIQA + individuals in various settings are reluctant to disclose their concerns to a health worker and given that they often constitute highly networked social groups [35], the use of peers in delivering mental health interventions appears particularly relevant. Interventions delivered by peers aimed at reducing HIV-related risk behaviours have been found to be highly effective among LGBTIQA + population in Nepal [36] and other LMICs [37]. However, based on the latest scoping review of global literature [31], the use of peers to deliver PM + to improve mental health outcomes among LGBTIQA + populations has not yet been robustly evaluated.

In addition to limited evidence on peer delivery, several significant knowledge gaps persist. The long-term impacts of PM + on psychological distress remain inadequately established, as the majority of studies have concentrated on short-term outcomes [33]. Evidence regarding the degree to which PM + can enhance perceived social support among marginalized populations is also lacking. Furthermore, there is limited understanding of whether the integration of individual PM + counselling with additional group sessions provides greater benefits than individual PM + alone. The present study aims to address these gaps by assessing these innovations within the sexual and gender minority population in Nepal.

Moreover, there is a paucity of evidence on effective interventions that can reduce exposure to violence against LGBTIQA + populations in LMICs. Safety planning has been found to reduce the risk of harm and re-victimization among women experiencing violence when implemented within healthcare and community domestic violence services [38–40]. It is also relevant for LGBTIQA + people [41] but the approach has not been adapted and evaluated for this group. Evidence also suggests that psychosocial support combined with safety planning is effective in improving mental health outcomes and further reducing exposure to violence among women [42, 43]. This provides a conceptual basis for integrating and adapting similar multicomponent approaches to address the dual burden of violence and mental disorders among LGBTIQA + populations.

The SAATHI (Support, Advocacy, and Awareness for Trauma, Healing and Inclusion) intervention is designed to address psychological distress and violence exposure through multiple, complementary mechanisms. Drawing on cognitive-behavioural theory, the PM + components target psychological distress through behavioural activation (countering avoidance and withdrawal), cognitive reappraisal (challenging negative thought patterns), and enhanced problem-solving capacity (increasing sense of control and self-efficacy) [34]. For LGBTIQA + populations specifically, these mechanisms address not only general distress but also distress stemming from minority stress [44]. The problem-solving component, for instance, may help participants develop strategies for navigating discrimination, while cognitive reappraisal may counter internalized stigma and self-blame related to violence exposure. The SAATHI intervention also includes empowerment-based techniques and practices to strengthen participants’ sense of autonomy and self-efficacy. Motivational interviewing techniques have been added to support the mobilization of the participants’ own resources for change, assuming that eliciting participants’ own reasons for change is more effective than prescribing specific methods or trying to persuade them. The safety planning component aims to equip clients with strategies to both feel safer and to take action to minimize exposure to violence. We expect that participants who receive the SAATHI intervention will experience less re-victimization than participants in the control arm. We hypothesize that reduced exposure to violence and increased behavioural activation, cognitive reappraisal, and problem-solving capacity will contribute to lower psychological distress and improved self-compassion after 12 months of intervention. The aim of the additional group sessions will be to strengthen social support among clients who may feel isolated and lonely due to societal marginalization and family exclusion. We anticipate that these meetings will create awareness among the clients that others have similar experiences as them and they are not alone, and that this will build stronger social connections and foster more positive feelings about their LGBTIQA + identity, helping to reduce the effects of hostile and threatening environments. We further expect a reduction in psychological distress and symptoms of post-traumatic stress, improved functioning and an increase in self-compassion, self-efficacy related to escaping violent relationships due to the individual augmented PM + counselling and increased use of community resources, social support and LGBTIQA + identity due to the addition of the group sessions compared to individual counselling alone.

### Overall objective

To implement and evaluate the feasibility, acceptability, and effectiveness of a SAATHI intervention in reducing exposure to violence and psychological distress among LGBTIQA + people in Nepal.

### Specific objectives

1. To determine the effectiveness of the augmented PM + with safety planning, empowerment, and social support activities delivered by peer advocates in reducing exposure to violence and psychological distress among LGBTIQA + people in Nepal.
2. To adapt augmented PM + with safety planning intervention to the Nepali context, aiming to reduce exposure to violence and psychological distress among LGBTIQA +.
3. To determine the feasibility of peer administration of augmented PM + with safety planning among LGBTIQA +.
4. To assess the foundational helping and PM + competencies of LGBTIQA + peers delivering the SAATHI intervention.
5. To explore participants’ and peer counsellors’ experiences and the opportunities and challenges encountered during delivery of the SAATHI intervention.

## Methods

### Trial design with patient and public involvement

Building on our longstanding history of conducting research with LGBTIQA + populations, we developed a multicomponent intervention titled SAATHI, which translates to ‘peer’ or ‘friend’ in Nepali. The SAATHI trial will serve as a neutral and culturally relevant label for the intervention, facilitating communication with other research teams, study participants, and relevant stakeholders.

This three-armed randomized controlled trial (RCT) adopts a superiority, multicentre parallel-group design. This study protocol is reported in accordance with the Standard Protocol Items: Recommendations for Interventional Trials (SPIRIT) 2025 statement and checklist (Supplementary file 1). 

Following a community-based participatory research (CBPR) approach [45], the project builds on equitable collaboration with affected community members throughout the research process. The study was conceived together with the umbrella organization for LGBTIQA + in Nepal, the Blue Diamond Society. BDS has been a key advocate for Nepal’s LGBTIQA + communities since its founding in 2001. Their work spans health promotion, HIV awareness campaigns, human rights advocacy, and legal support services. Beyond that, BDS documents violations of rights, provides legal counselling, runs crisis shelters, and even supports income generation programs for marginalized LGBTIQA + individuals. This organization suggested the sites for the study.

We have followed the Castro-Barrera framework [46] to culturally adapt the SAATHI intervention to the target group. First, evidence was gathered on interventions targeting mental disorders and violence prevention among vulnerable populations, including LGBTIQA + individuals. This was done through a targeted literature review. Subsequently, we conducted a formative assessment from September to October 2024 involving 13 in-depth interviews with LGBTIQA + individuals, 35 key informant interviews with community leaders, mental health specialists, public health experts, and non-governmental organization representatives, and five focus group discussions with LGBTIQA + individuals (30 participants in total, with six participants per focus group). Semi-structured interview guides were tailored to each target audience. This process explored cultural preferences, needs, barriers, and common stressors among the LGBTIQA + population.

The formative assessment and consultation meetings with subject experts guided the development of tools and forms, as well as the combination, adaptation, and augmentation of the SAATHI intervention. Safety planning content was revised to reflect local contexts (omitting irrelevant items such as having car keys in safe place). The session flow was adjusted, and case examples were contextualized for this population. Interviews with these individuals, particularly community representatives, highlighted the need for case vignettes that not only depict adversity but also highlight resilience and resourcefulness in challenging situations. The draft manual was reviewed by community leaders and selected LGBTIQA + participants in the formative assessment to ensure culturally appropriate language and reading levels. Considering the varying capacities of participants, recommendations included preparing flip charts summarizing key session activities.

The assessment also addressed whether peer counsellors should share the same gender or sexual orientation as the clients. Based on inputs from respondents, this was made optional. Thus, after randomization, participants may choose whether they prefer a helper with the same or a different gender or sexual orientation.

This process has ensured meaningful participation of community members at every stage of the project. The benefits of adopting the CBPR approach are that it develops a sense of ownership among community members and builds the capacity of LGBTIQA + individuals to generate locally relevant evidence to address their own challenges. During the formative phase, consultations also took place with mental health professionals, public health experts, and service providers to shape SAATHI into a culturally appropriate and contextually grounded intervention.

### Trial setting

The study is ongoing across seven districts of Nepal (Kathmandu, Lalitpur, Chitwan, and Makwanpur from Bagmati Province and Sarlahi, Dhanusha, and Parsa from Madhesh Province). We chose the study districts based on two factors: the size of local LGBTIQA + populations and the strength of community networks in those areas. A dedicated facility has been set up in each district with private rooms for screening, assessments, and counselling sessions, as well as separate waiting areas to ensure confidentiality and accessibility. Since Kathmandu and Lalitpur are neighbouring districts and there is frequent cross-movement of the residents between these two districts, one facility serves both.

### Eligibility criteria

The eligibility criteria for trial participants are as follows: self-identify as LGBTIQA + individual, aged 18–55 years, history of any form of violence in the previous 12 months, and experiencing psychological distress (a score of ≥ 3 on the General Health Questionnaire-12 scale). The exclusion criteria are as follows: suicidal ideation, self-report or suspected to have a severe mental disorder such as psychosis, alcohol or drug use dependence, severe intellectual disability, or plans to leave the study district within a few months. An additional exclusion criterion is participation in concurrent structured psychotherapy or similar psychological therapies, including cognitive behavioural therapy, interpersonal psychotherapy, or other structured trauma-focused psychotherapies.

The peer advocates or peer counsellors are LGBTIQA + members aged 18 + with basic literacy; motivation to provide psychosocial support; commitment to community service; and prior experience from other LGBTIQA + programs.

### Intervention and comparator

Participants in this trial will be enrolled into one of three study arms: (a) the Enhanced Usual Care (EUC) arm; (b) the SAATHI-Individual counselling (SAATHI-I) arm; and (c) the SAATHI-Individual plus Group counselling (SAATHI-IG) arm. Participants in the control arm will receive enhanced usual care, which includes access to information about local (formal and informal support) violence prevention and mental health resources within their community that they can reach out to if needed. If they express suicidal ideation, they will be offered accompanied referral to a mental health specialist for immediate care. They will also receive information about different types of violence, the mental health consequences of experiencing violence, and general stress reduction strategies such as engaging in physical activity, avoiding harmful habits (smoking and alcohol use), ensuring adequate rest, and maintaining a balanced diet. Participants will be informed that mental disorders are treatable, and violence is preventable, and toll-free hotlines are available for support if they experience violence or mental health crises. All participants, independent of arm, who report that they are in danger and do not have any friends or relatives to stay with, will be provided with support and shelter in collaboration with local organizations including Blue Diamond Society and its sister organizations. All this information will be documented in a leaflet and distributed to participants in the three arms of the trial, regardless of allocation.

#### Intervention structure, delivery, and providers

The SAATHI intervention is an adapted psychosocial program based on the core elements of PM + [32] and individualized safety planning [39]. The intervention also includes empowerment exercises [47–49] to strengthen participants’ sense of autonomy and self-efficacy given the widespread stigma, discrimination, and social rejection associated with same-sex sexual behaviour in Nepal. It aims to lessen the psychological effects of trauma by helping participants reframe stressful situations, develop better coping strategies (including slow breathing, mindfulness, managing problems, social support, ‘get going, keep doing’, safety planning), and strengthen self-regulation, self-empathy, and self-esteem. The use of motivational interviewing [50] techniques to mobilize the participants’ own reasons for change not only aims to increase adoption of the new strategies, but also ensures that every session will be delivered in a manner that respects and acknowledges the participants’ unique identity and lived experience.

The SAATHI-I intervention consists of six weekly individual counselling sessions, each lasting 60–90 min. The sessions are delivered face-to-face in a private room at the study sites. A comprehensive SAATHI manual has been developed, including detailed descriptions of the topics to be discussed with population-specific case vignettes, table flip charts—with a concise summary of the key components of each session to support counselling sessions, safety plan templates, and self-monitoring forms to support the practice of the SAATHI strategies. Supplementary file 2 summarizes the SAATHI-I components. The self-monitoring forms promote the concept of implementation intentions [51, 52]—encouraging clients to apply the strategies in their daily lives, particularly in contexts where they feel comfortable. Evidence suggests that the use of implementation intentions increases the chance of adopting desired habits and achieving targeted outcomes.

Trusted family members, partners, or friends may be included in orientation and basic skills sessions to help clients apply the key strategies outside the sessions, but only with client’s consent. They will not attend sessions that address violence, stress management, problem solving, or safety planning. The client and peer advocates will jointly decide whether the presence of a trusted person promotes comfort and confidentiality or poses any concerns.

The peer advocates who deliver the sessions are advised not to conduct more than four sessions per day to prevent emotional burnout. Additionally, they are encouraged to take a 30-min break between sessions to rest. Peer advocates will receive a monthly salary for their role as helpers in the study.

Participants who are allocated to the SAATHI individual plus group (SAATHI-IG) arm will also be invited to attend monthly group meetings (6–10 participants in each group) to strengthen social support. These group meetings will tentatively run for 60 to 90 min. During the sessions, participants can share their experiences with the SAATHI strategies (see Supplementary file 2), discuss the benefits and challenges of applying them in their daily life, and offer peer support. The group sessions aim to help participants maintain strategies that reduce psychological stress.

#### Monitoring of fidelity and adherence

Intervention fidelity will be monitored by trained study team members (public health and mental health professionals) using standardized session checklists. At the start of the intervention period, they will conduct structured observations of all six sessions for two clients per peer advocate. They will support peer advocates in implementing the sessions according to the manual, address and document any implementation queries they may have. The study team will monitor scheduled intervention sessions throughout the intervention period, with intensive monitoring and debriefing during the initial phase and needs-based support in later phases. In addition, exit client interviews after all six sessions are completed will be conducted by another peer advocate or a field researcher to assess what they learned. Information on session duration will be regularly reviewed for each peer advocate to check that it is within the anticipated range for what is needed to cover the prescribed content.

To encourage adherence to the allocated treatment, attendance of individual and group sessions will be recorded, and follow-up calls will be made to remind participants of upcoming sessions. Participants who miss scheduled sessions will be followed up by peer advocates within 48 h and the session will be rescheduled at the earliest convenience of the participant.

Peer advocates will review participants’ adherence to the SAATHI strategies through self-monitoring forms during individual sessions and will provide guidance as needed using motivational interviewing-informed techniques.

#### Criteria for discontinuation and concomitant care

The SAATHI intervention will be discontinued if a participant decides to withdraw. In addition, the SAATHI delivery may be modified by shortening sessions, pausing delivery, or adjusting the pacing of the content in response to participants’ needs, e.g. in cases of acute distress, fatigue, emotional overwhelm, or low processing capacity. SAATHI sessions will be postponed if participants exhibit active suicidal ideation with intent or an escalating risk of violence. Such participants will be referred immediately to specialist care.

Participants in the SAATHI trial may access usual primary care, emergency mental health services, and informal community support activities led by community-based organizations, such as awareness campaigns, meetings, and workshops on the health and rights of LGBTIQA + population. Participation in psychoeducational workshops or general support groups that are not based on a structured psychotherapy model will also be permitted.

#### Training of peer advocates in the SAATHI Individual Counselling (SAATHI-I) and SAATHI Individual Counselling plus Group Meetings (SAATHI-IG) packages

The training sessions were facilitated by a multidisciplinary team, including a psychiatrist, public health professionals, and community leaders with substantial academic and field experience in mental health and violence prevention, particularly in work with vulnerable and marginalized populations.

The 10-day program used a structured approach grounded in experiential learning. It blended participatory lectures and case discussions with live demonstrations, role-playing, and guided practice interviews. The training aimed to build both conceptual knowledge and hands-on skills among peer advocates through practice and expert feedback. Peer advocates learned to apply the adapted PM + strategies, create individualized safety plans, and practise motivational interviewing. The PM + intervention was adapted by adding dedicated session on safety planning and self-empowerment and by integrating self-acceptance and self-kindness content [53] across sessions. In addition, implementation intention strategies were incorporated to encourage clients to plan specific time during the week to practise SAATHI strategies, document their practice using strategy-specific forms, and review and discuss this with peer advocates in subsequent sessions.

Beyond these core skills, the training also covered empowerment-based techniques and practices for autonomy and self-efficacy. Peer advocates were trained in the use of different strategies from motivational interviewing to help them build rapport with clients during counselling sessions and strengthen clients’ motivation to change. The training covered the use of open-ended questions, affirming and supporting clients’ statements of understanding and intention to change, and practising reflective listening to draw out clients’ own arguments for change rather than presenting these to them. The peer advocates were also trained to use summarizing techniques to capture the key points of the discussion, link related topics, and guide transitions within the conversation.

To ensure peer advocates could deliver counselling well, we trained them using the Enhancing Assessment of Common Therapeutic factors (ENACT) competency tool from the Ensuring Quality in Psychosocial and Mental Health Care (EQUIP) platform [54, 55]. Core interpersonal skills such as verbal and non-verbal communication, emotional attunement, the ability to explain confidentiality, and risk detection were assessed with standard observation checklists both before and after the sessions that introduced the related content. Specifically, the Nepali version of ENACT items 1–7 from the EQUIP platform was used. For items 8–16 (about PM + Competency) we had them translated and back translated between English and Nepali by subject matter experts and linguistic specialists to ensure semantic and conceptual equivalence.

Our EQUIP-based training also emphasized trauma-informed care, ethical engagement, and cultural responsiveness. We prepared peer advocates to apply these principles in high-risk contexts like supporting participants facing chronic violence and stigma. A key part of this was integrating safety planning and harm reduction strategies specifically designed for ongoing interpersonal or community violence. Peer advocates practised assessing safety risks, developing personalized safety plans appropriate for LGBTIQA + individuals, and navigating local referral systems for protection and support for their clients.

To consolidate learning, each day concluded with structured reflection sessions, in which peer advocates shared experiences and challenges and received feedback on their practice. Role plays based on challenging case vignettes were integrated throughout the training to build confidence and facilitate practical application of the SAATHI intervention strategies.

To prepare peer advocates for the field implementation, all received an orientation on the SAATHI intervention manual. The training also covered essential documentation procedures, such as using adherence checklists, maintaining session registers, entering Psychological Outcome Profiles (PSYCHLOPS) data into the Research Electronic Data Capture (REDCap) tool, and adhering to debriefing protocols. These quality assurance measures are designed to support the fidelity of intervention delivery across implementation sites.

#### Training of peer advocates in EUC arm

Peer advocates in the control arm completed a structured, 2-day training. Its goal was to ensure the ethical, accurate, and participant-centred delivery of EUC. We equipped advocates with the knowledge and communication skills needed to share mental health and violence prevention information, respond with sensitivity to participants’ needs, and refer clients to mental health care when needed.

The program used a participatory format, blending interactive lectures with case-based discussions, group exercises, and guided demonstrations. It started with orientation sessions that covered the study’s objectives, the role of EUC, and key ethical principles like confidentiality, voluntary participation, and non-coercive communication.

A key part of the training focused on how to clearly explain the scope and limitations of EUC. Facilitators employed pre- and post-test questions to gauge knowledge gains. They also observed role plays performed by the peer advocates and provided feedback to ensure that all peer advocates met the minimum quality standards for dos and don’ts related to ENACT items 1–7, particularly when informing participants about the EUC or administering PSYCHLOPS. Finally, the training introduced procedures for documenting referrals and follow-ups using EUC-specific recording templates.

### Outcomes

#### Primary outcomes

The primary outcomes of the study are psychological distress and experience of violence 12 months after baseline (Table 1). Psychological distress will be measured using the GHQ-12 [56]. The GHQ-12 consists of 12 items and will be scored using the bimodal scoring method. It assesses symptoms over the past 2 weeks, with a total score ranging from 0 to 12, where a higher score indicates greater levels of psychological distress.

**Table 1:** Description of primary outcome measures, measurement tools, scoring, and time of measurement

Outcome/sub-outcomes	Measurement tool	Description and domains	Scoring	Time of measurement (since baseline)
Psychological distress	GHQ-12	Psychological distress in the past 2 weeks; includes symptoms such as anxiety, depression, and social dysfunction	4-point Likert scale ('less than usual' to 'much more than usual'); scored using bimodal method (0–0–1–1); total score 0–12	12 months
Experience of any violence	Modified Women’s Health questionnaire*	Any physical, sexual, and psychological violence or controlling behaviours experienced in the past 3 months	Binary (1 = any act; 0 = none)	12 months

*GHQ-12* General Health Questionnaire—12 item

*Modified WHO Multi-country Study Questionnaire on Women’s Health and Domestic Violence

Experiences of violence will be measured using a modified version of the WHO multi-country study on women’s health and domestic violence questionnaire [57]. This tool assesses exposure to physical, sexual, and psychological violence and controlling behaviours. This binary outcome captures whether the participants report any violence or controlling behaviours in the past 3 months.

#### Secondary outcomes

Psychological distress will also be assessed at 7 and 18 weeks to evaluate the short and intermediate impact of the intervention (Table 2). Whereas experiences of violence (type-specific violence and severity/high-intensity violence) will be assessed at 18 weeks and at 12 months. Each type of violence will be analysed as a separate binary outcome based on whether the participants report any act within the category. Severity or high-intensity violence will be assessed using established criteria [58], which classify several physical acts, repeated less-severe acts, frequent emotional or controlling acts, multiple emotional or controlling acts occurring once or a few times, and all sexual violence as indicators of high-intensity violence.

**Table 2:** Description of secondary outcome measures, measurement tools, scoring, and time of measurement

Outcome	Measurement tool	Description and domains	Scoring	Time points*
Psychological distress	GHQ-12	Psychological distress over the past 2 weeks (anxiety, depression, and social dysfunction symptoms)	4-point Likert; bimodal scoring (0–0–1–1); total score 0–12	7 weeks, 18 weeks
Experience of physical violence	Modified Women’s Health Questionnaire**	Slapping, pushing, hair pulling, hitting, kicking, dragging, choking/burning, or threat/use of weapon	Binary (1 = any act; 0 = none)	18 weeks, 12 months
Experience of sexual violence	Modified Women’s Health Questionnaire	Forced sex, degrading/humiliating sexual acts or sex due to fear	Binary (1 = any act; 0 = none)	18 weeks, 12 months
Experience of psychological violence	Modified Women’s Health Questionnaire	Insults, belittling, humiliation, intimidation, or threats to harm participant or someone they care about	Binary (1 = any act; 0 = none)	18 weeks, 12 months
Experience of controlling behaviours (partner or family)	Modified Women’s Health Questionnaire	Restricting the participant’s social contact, surveillance, jealousy, restricting healthcare access, or blocking income-generating activities	Binary (1 = any act; 0 = none)	18 weeks, 12 months
Experience of severe/high-intensity violence	Modified Women’s Health Questionnaire	1 = Yes if participant reports ≥ 1 severe physical act, OR ≥ 2 less-severe physical acts, OR ≥ 1 emotional/controlling act “many times,” OR ≥ 3 different emotional/controlling acts “once/few times,” OR ≥ 1 sexual violence act. 0 = No if none of these criteria are met	Binary (1 = high-intensity; 0 = not high-intensity)	18 weeks, 12 months
PTSD symptoms	PTSD Checklist for DSM-5 (PCL-5)	20-item self-report of PTSD symptoms in past week	5-point Likert (0–4: 'not at all' to 'extremely'); total score 0–80	7 weeks, 12 months
Daily functioning	WHODAS 2.0, 12-item	Six domains of functioning (cognition, mobility, self-care, getting along, life activities, participation) in past 30 days	5-point Likert ('no difficulty' to 'extreme difficulty/cannot do'); total score 0–48	18 weeks, 12 months
Self-esteem	Modified Collective Self-Esteem Scale	16 items related to current attitudes, feelings, perceptions about belonging to the LGBTIQA + community	7-point Likert ('strongly disagree' to 'strongly agree'); total score 16–112	18 weeks, 12 months
Self-compassion	Self-Compassion Scale—Short Form	12 items related to current self-kindness, self-judgment, common humanity, isolation, mindfulness	5-point Likert scale ('almost never' to 'almost always'); total score 12–60	18 weeks, 12 months
Perceived social support	Modified Social Support Survey	5 items related to availability of emotional, informational, tangible, affectionate, and positive social interaction support in past 4 weeks	5-point Likert scale ('none of the time' to 'all of the time'); total score calculated as mean or sum, 5–25	12 months
Use of personal safety strategies	Modified Safety Planning Checklist	Use of practical safety measures (safe place, essential items, emergency plan, contact list) in response to potential violence	Binary (yes/no) for each item; descriptive summaries	18 weeks, 12 months
Use of community resources	Modified Women’s Health Questionnaire	Use of violence prevention/support resources reported at follow-up assessments	yes/no;	18 weeks, 12 months
Satisfaction with community resources	Modified Women’s Health Questionnaire	Satisfaction level with violence prevention/support resources reported at follow-up assessments	5-point Likert ('not satisfied at all' to 'very satisfied')	18 weeks, 12 months
Psychological outcomes profile	PSYCHLOPS	Psychological distress across problem severity, functioning and well-being over the past week	6-point Likert scale, each item scored 0–5; total score 0–20; plus, qualitative responses	7 weeks, 18 weeks

*PTSD* Post-traumatic stress disorder; *PCL-5* Post-traumatic Stress Disorder Checklist for DSM-5, *DSM-5* Diagnostic and Statistical Manual of Mental Disorders, Fifth Edition, *WHODAS 2.0* WHO Disability Assessment Schedule 2.0, *PSYCHLOPS* Psychological Outcome Profiles

^*^When multiple time points are listed for an outcome, each time point represents a distinct secondary outcome and will be analyzed separately

^**^Modified WHO Multi-country Study Questionnaire on Women’s Health and Domestic Violence

Post-traumatic stress disorder (PTSD) symptoms will be measured using the PTSD Checklist for DSM-5 (PCL-5) [59], a 20-item self-report instrument. It assesses the presence and severity of PTSD symptoms experienced during the past week, rated on a 5-point Likert scale ranging from ‘not at all’ to ‘extremely’. Total scores range from 0 to 80, with higher scores indicating greater PTSD symptom severity. PCL-5 scores will be measured at 7 weeks and 12 months.

Daily functioning will be assessed using the WHO Disability Assessment Schedule (WHODAS 2.0, a 12-item version) [60], which measures functional impairment across six domains (cognition, mobility, self-care, getting along with people, life activities, and participation in society) over the past 30 days. Responses are rated on a 5-point Likert scale ranging from ‘no difficulty’ to ‘extreme difficulty/cannot do’. Total scores range from 0 to 48, with higher scores indicating greater functional impairment. WHODAS scores will be assessed at 18 weeks and 12 months after baseline.

Self-esteem will be measured using the Modified Collective Self-Esteem Scale [61], which assesses participants’ attitudes, feelings, and perceptions regarding their belonging to the LGBTIQA + community. The 16-item scale uses a 7-point Likert response format ranging from ‘strongly disagree’ to ‘strongly agree’. Total scores range from 16 to 112, with higher scores reflecting more positive evaluation of, and stronger identification with, the group. Self-esteem will be measured at 18 weeks and at 12 months.

Self-compassion will be assessed using Neff’s Self-Compassion Scale—short form [62]. This 12-item measure evaluates components including self-kindness, self-judgement, common humanity, isolation, and mindfulness. Items are rated on a 5-point Likert scale ranging from ‘almost never’ to ‘almost always’, producing a total score from 12 to 60, with higher scores indicating greater self-compassion. Self-compassion scores will be assessed at 18 weeks and at 12 months.

Perceived social support will be assessed using the Modified Social Support Survey 5-item version (MSSS-5), which evaluates the availability of various types of support, including emotional/informational, tangible, affectionate, and positive social interaction, during the past 4 weeks. Responses are provided on a 5-point Likert scale ranging from ‘none of the time’ to ‘all the time’, producing a final score between 5 and 25, where higher scores indicate greater social support. The assessment will be made at 12 months.

Personal safety strategies will be measured using a Modified Safety Planning Checklist, which assesses participants’ use of personal safety measures in response to potential violence. Items cover practical activities such as securing a safe place to stay, keeping essential documents or items (e.g. medicines, cash, pair of extra clothes), preparing an emergency safety plan, and maintaining an emergency contact list. The outcome will be analysed as total checklist scores at 18 weeks and at 12 months after baseline, with higher scores indicating more safety strategies used.

Use and satisfaction with community resources for violence prevention and support will also be assessed. Use will be measured as a binary variable (yes/no to any use of services), and satisfaction will be measured on a 5-point Likert scale ranging from ‘not satisfied at all’ to ‘very satisfied’.

The Psychological Outcomes Profile (PSYCHLOPS) [32, 63] of the participants will be assessed using a self-reported measure designed to capture progress from the participants’ perspective by evaluating four questions related to problems, functioning, and well-being. Responses are provided both as free text narratives and on an ordinal six-point scale. Each question is scored from 0 to 5, producing a maximum total score of 20. The tool also includes an item assessing suicidal thoughts. Follow-up assessments will be made at 7 weeks and at 18 weeks after baseline assessments. Follow-up assessments using PSYCHLOPS will also include a question on participants’ current overall perception of change, rated from ‘feeling much better’ to ‘feeling much worse’, compared with their state prior to participating in the SAATHI intervention.

The information about outcome measures is summarized in Tables 1 and 2.

### Harms

The following harms will be monitored for this trial: increased psychological distress, worsening of existing mental health symptoms, new or intensified suicidal ideation, heightened fear or anxiety, and increased exposure to violence associated with participation in the intervention or research procedures. Studies have shown that there are no observed increases in serious adverse events, such as an increase in suicidal thoughts, associated with PM + when it is delivered by trained and supervised non-specialist helpers [31]. However, PM + will not be offered to high-risk individuals such as those with severe mental disorders or those at heightened risk of suicide, as the PM + is designed to address mild to moderate psychological distress.

Some safety strategies, including disclosure to informal networks or seeking help from formal institutions, may involve risks in certain contexts [64]. These risks can include escalation of violence or retaliation when involving others due to conflicts with the norms of family privacy, particularly when institutional support responses are ineffective. Evidence also indicates that safety planning may be of limited value where access to psychosocial services, housing assistance, and legal protection is constrained, which can leave survivors at continued risk [65]. Ensuring the availability of adequate support services is therefore necessary when promoting safety planning strategies.

As part of the individual counselling and the group sessions, participants will be invited to share and reflect on adversities, as well as explore possible solutions. This process may lead to increased psychological stress or emotional discomfort. Harms will be monitored systematically using tools such as PSYCHLOPS suicidal ideation item during counselling sessions or at follow-up assessments. Any adverse events will be documented using a standardized adverse event reporting form. Any serious events (suicide attempts, hospitalization for mental health reasons, escalation in the risk of violence, or any event that poses substantial danger to the participant) will be reported to the Data and Safety Monitoring Board for review and guidance.

This protocol is designed to safeguard all SAATHI participants. In cases of suicide risk, the study team will provide immediate referral to a mental health specialist, with the participants’ agreement, or link them to local support services in situations of risk of interpersonal violence.

There is also a potential risk of psychological distress or emotional exhaustion among peer advocates. This may arise from repetitive exposure to traumatic experiences shared by participants, as well as the peer advocates’ own daily challenges in a highly stigmatized environment. To reduce this risk, peer advocates will receive structured support. This will include regular debriefing sessions with mental health professionals, limits on the participants they work with each day, scheduled self-care breaks between sessions, and training in mindfulness and slow breathing techniques. In cases where peer advocates’ personal well-being is affected, individual counselling support will be made available. Burnout assessment will also be included in routine monitoring visits.

### Participant timeline

**Fig. 1:**
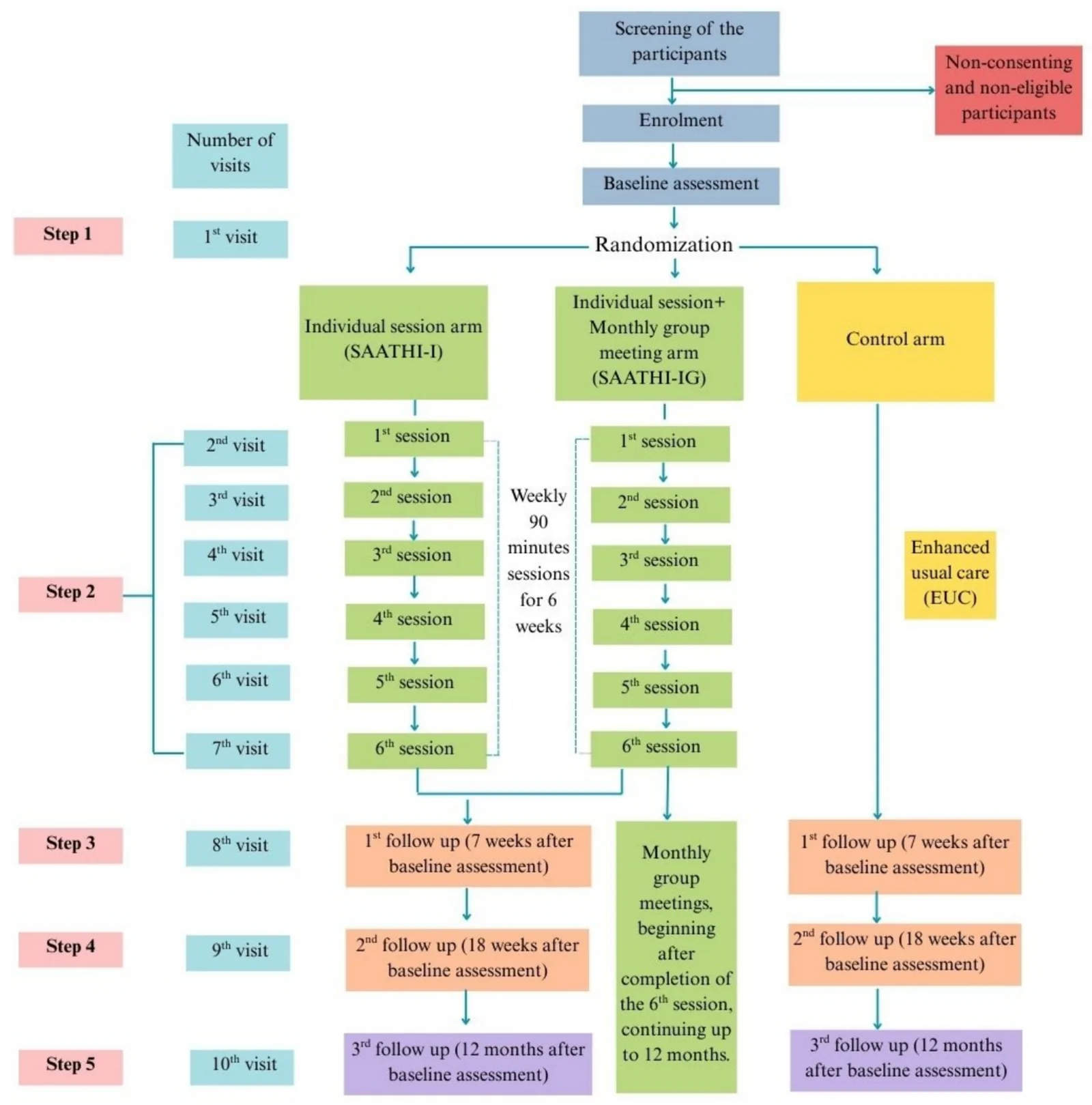
Schedule of enrolment, intervention delivery, and follow-up visits across study arms

Figure 1 demonstrates the sequential steps of the SAATHI trial, beginning from screening and enrolment, followed by intervention delivery and subsequent follow-up assessments. Follow-up assessments will be conducted at baseline, and at 7 weeks, 18 weeks, and 12 months after baseline for all study arms. The schedule of enrolment, interventions, and assessments is outlined in Table 3.

**Table 3:** Schedule of enrollment, interventions, and assessments

	Trial period					
Timepoint	Enrollment (-t1)	Allocation (0)	Intervention period (weeks 1–6)	7 weeks	18 weeks	12 months
Enrollment						
Outreach/Invitation	X					
Eligibility screen	X					
Informed consent	X					
Baseline Assessment		X				
Allocation/Randomization		X				
Interventions						
EUC		X				
SAATHI-I		X	X			
SAATHI-IG*		X	X	X	X	X
Assessments (Quantitative)						
Demographics/baseline characteristics	X	X				
Psychological distress						X
Experience of violence						X
Secondary outcomes**				X	X	X
Adherence/fidelity/safety						
Attendance tracking (individual + group sessions)			X			
Intervention fidelity checks (session, checklist; observation/debriefing)						X
Adverse event monitoring/referral if needed	X	X	X	X	X	X
Process evaluation (Qualitative)						
Interviews with participants and peer advocates			X	X	X	X

-t1 (enrolment): screening/consent prior to baseline assessment; 0 (Allocation): baseline assessment and randomization

^*^Group sessions begin after individual sessions and continue through follow-up period)

^**^Details of assessment time points for secondary outcomes are provided in Table 2

### Sample size

The SAATHI trial will enrol at least 960 participants (320 per arm). This sample size is estimated to provide 90% power to detect a standardized mean difference of 0.27 in psychological distress between the EUC arm and the SAATHI-I arm and between the SAATHI-I arm and the SAATHI-IG arm at 12 months after enrolment. Other parameters used in the sample size calculation include a standard deviation of 2.6 units [66], a two-sided α of 0.05, and a maximum assumed attrition rate of 10%. This standardized effect corresponds to an absolute mean difference of approximately 0.7 points on the GHQ-12 bimodal scale. The assumed effect size is conservative and is consistent with the range of effects reported in previous evaluations of PM + [67, 68] and with methodological work on clinically relevant effect size for psychotherapy [69].

This sample size is also sufficient to detect a 25% reduction in the proportion of participants reporting exposure to violence after receiving the PM + counselling (from 60% in the control arm to 45% in the SAATHI-I arm) at 12 months, with at least 90% power. To ensure adequate power for the planned secondary outcomes, the final planned enrolment was increased to at least 354 participants per arm (total *N* = 1062).

### Recruitment

Peer advocates will invite individuals from their personal and community networks to participate in the study. Study participants will also be encouraged to refer their friends, relatives, and partners. Given that LGBTIQA + populations are highly networked, this purposive snowball sampling strategy is expected to reach a substantial number of participants. In addition, the peer advocates will use social media platforms to disseminate information. This includes a study-specific Facebook page for each site, where contact details of peer advocates and study sites will be shared. Peer advocates will also distribute study messages through other platforms such as Viber, WhatsApp groups, and online dating sites. All messaging will follow privacy and confidentiality guidelines. Information about the study will be further shared through social and cultural gatherings, as well as via local mass media, particularly community radio (FM stations). Peer advocates will conduct outreach at known gathering places (often referred to as ‘hotspots’) frequented by LGBTIQA + communities to promote the study and encourage participation.

### Randomization and blinding

Participants are randomly assigned to one of the three study arms using simple randomization, with equal allocation ratios (1:1:1 ratio). To ensure impartiality, an independent researcher (not involved in delivering intervention sessions or assessing outcomes) at the central office handles the randomization process. The allocation sequence will be concealed from the peer advocates by ensuring that the peer advocates are informed of the participant’s assignment only after enrolment is completed. The assigned arm will be communicated to the responsible peer advocate by the central study team using the participants’ study code via secure mobile messaging or a telephone call.

Blinding of participants and peer advocates will not be implemented due to the nature of the intervention. The field researchers, who are not involved in delivering the SAATHI intervention, will be responsible for outcome assessments and will not have access to the randomization sequence. Although they may become aware of participants’ allocation during follow-up interviews or by working in the same building as the peer advocates, steps will be taken to minimize detection bias. Measures will include standardized procedures such as structured questionnaires, scripted instructions, neutral delivery of questions, and training of field researchers in assessment procedures. However, the new independent outcome assessors responsible for the final assessment of the primary outcomes will be blinded.

Unblinding of the outcome assessors will be permissible only if knowledge of the assigned intervention is required to ensure participant safety or to enable appropriate clinical management following a serious adverse event. Any request for unblinding will be made by the principal investigator to the researcher responsible for maintaining the allocation list. The allocation will be disclosed only for the individual participant concerned and only to the minimum personnel required. The date, reason, and individuals involved in any unblinding will be documented.

### Data collection methods

The quantitative questionnaires for the randomized controlled trial include screening, baseline, and follow-up instruments. These instruments include demographic questions and standardized validated tools, including the GHQ-12, WHO multi-country questionnaire for violence assessment, WHODAS 2.0, PCL-5, PSYCHLOPS, safety behaviour checklist, MSSS-5, Kristin Neff’s Self-Compassion Scale, and the Modified Collective Self-Esteem Scale. The validated tools were adapted using standard recommended approaches for cross-cultural adaptation of self-reported measures [70]: First, native speakers with relevant expertise translated the tools from English into Nepali, Bhojpuri, and Maithili. Study staff reviewed the translations and resolved discrepancies. Independent bilingual individuals then back translated the instruments into English. The study team compared the back translations with the originals and used the identified differences to refine and finalize the local language instruments. Language experts and community leaders subsequently reviewed the revised instruments to simplify terminology for lay understanding while preserving meaning. The complete questionnaire was pre-tested with 10 field researchers and pilot tested with 28 LGBTIQA + community members to ensure clarity and acceptability. Although the study team had already adapted GHQ-12, WHO multi-country questionnaire for violence assessment, WHO Disability Assessment Schedule 2.0, PCL-5, PSYCHLOPS, and a safety behaviour checklist for vulnerable populations in Nepal [71], we went through all the above steps again to make sure they were also a good fit for the LGBTIQA + community.

During the trial, trained field researchers with public health backgrounds will conduct face-to-face interviews using tablets at enrolment (baseline assessment), at 7 weeks (corresponds to 1 week after sixth session for the intervention groups), 18 weeks, and 12 months after the baseline assessment. Figure 1 illustrates the sequential steps from screening to intervention delivery and follow-up assessments. Interviews will be conducted in Nepali, Bhojpuri, and Maithili based on participant preference. Data collected through REDCap will be synchronized directly with a secure server at the central office. Monitoring and supervision of the field researchers (both scheduled and ad hoc) will take place weekly to bi-weekly during the initial phase and will transition to monthly intervals in later phases to maintain data quality.

To promote participant retention, multiple strategies will be implemented. Contact information, names of alternative contact persons, and preferred times for follow-up calls will be recorded to maintain communication. The central and field study teams will regularly review scheduled visits and follow-up timelines, with the central team providing oversight and coordination as needed. The field team will contact participants to provide timely appointment reminders for follow-up assessments. If a participant misses a follow-up appointment, they will be contacted to reschedule the visit. The study will provide incentives to cover transportation costs and incidental expenses (e.g. mobile data, basic refreshments) as compensation for participants’ time and effort.

#### Training of outcome assessors

The 5-day training of field researchers used a blended learning approach combining lectures, demonstrations, role plays, and hands-on practice with both paper-based and digital tools. It was facilitated by the research team in collaboration with experts with academic backgrounds and applied experience in public health, research methods, and mental health from other organizations. The training covered a range of methodological and ethical topics. Core components included digital data collection using REDCap, participant eligibility screening, administration of standardized assessment tools, referral protocols, and procedures for baseline and follow-up assessments. Given the sensitive nature of the study, field researchers were also trained in foundational helping skills such as respectful and inclusive verbal and non-verbal communication, maintaining confidentiality, rapport building, demonstrating sensitivity to participants’ lived experiences, empathy and exploring and normalizing feelings and assessing psychological harm. The field researchers were shown role play videos illustrating helpful and unhelpful behaviours related to ENACT items 1–7 developed by EQUIP. Role plays and supervised field-testing prepared field researchers to conduct interviews with accuracy and empathy.

### Process evaluation

The process evaluation will include observations and interviews conducted by the central study team. Monitoring visits will assess fidelity to the manual and the quality of counselling sessions. Semi-structured interviews will explore how participants and peer advocates experience the intervention and interpret it within their personal and cultural contexts. Participants will be interviewed about the counselling sessions, the usefulness of the safety planning and empowerment exercises, and the influence of group activities on social support. Peer advocates will be interviewed about their experiences and barriers during intervention delivery. In addition, to complement the quantitative ratings of peer advocates’ competencies, in-depth interviews will be conducted with the peer advocates responsible for delivering the sessions. These interviews will explore their experiences of applying the competencies in real-world settings.

The following research questions will be examined:

- What do participants and peer advocates experience as facilitators and barriers for change related to the specific components of SAATHI intervention and the social and cultural setting it is conducted within?
- How participants and peer advocates narrate and give meaning to change processes experienced by participants in relation to the SAATHI intervention (including both positive changes and possible adverse reactions)?
- How does being a client or peer advocate influence their self-definition in social relationships and narrative constructions of identity?

Data collectors with public health background stationed at central office will conduct in-depth interviews with approximately 20 clients and 20 peer advocates—the exact number will depend upon when saturation in central themes is reached. We will also conduct 3–6 focus group discussions with peer advocates to facilitate collective constructions of meaning based on common experiences. We will conduct only individual interviews with participants to ensure confidentiality and to allow reflection upon how personal background and life context contribute to experiences with the intervention.

The study will also evaluate the peer advocates’ competencies both before and after training using standardized observation tools. This assessment involves structured ratings of behaviours demonstrated during the role plays. Each competency scale will be scored on a four-level scale. Level 1 represents unhelpful or potentially harmful behaviour. Level 2 indicates the absence of skills or only partial demonstration of basic skills. Level 3 reflects the consistent demonstration of all basic helping skills. Level 4 reflects the demonstration of all basic helping skills together with one or more advanced skills. In addition, peer advocates’ practice will be assessed through direct observations of all six counselling sessions for two clients per peer advocate. A standardized checklist developed by EQUIP platform will be used during these observations. Competency will be assessed at three time points: prior to training and immediately post-training using standardized role plays, and during service delivery through direct observation of two counselling sessions, with the most recent observed session used for competency scoring. For each assessment, competency item scores will be averaged to generate mean scores for basic helping scores and PM + specific delivery skills, as well as an overall competency score. Peer advocates will be considered competent for service delivery if they achieve a score of 3 or higher on at least 80% of items and demonstrate no behaviours rated as potentially harmful. As part of the process evaluation, any behaviours rated as potentially harmful during post-training role plays or observed counselling sessions in the field will be shared with the peer advocates to support feedback and skill improvement.

### Data management

All quantitative data will be directly entered into REDCap at the point of collection by field researchers and peer advocates (who will be responsible for entering PSYCHLOPS data only). To minimize data entry errors, the system incorporates built-in validation rules, range checks, branching logic, and predefined legal values. Interim data exports will be encrypted and stored in a secure repository at the central office on a weekly basis. Access to collected data is restricted through role-based permissions and strong password requirements. The study team will conduct regular data quality checks (e.g. missing values, outliers, inconsistencies). Identified errors will be documented in a separate Excel sheet, including site and client codes, and shared with the field teams for resolution.

All qualitative data will be collected using digital audio recorders during in-depth interviews, key informant interviews, and focus group discussions. Informed consent for recording will be obtained prior to each interview. Audio recordings will be transferred to a secure server at the central office. Recordings will be deleted from the recording devices once the transfer is complete. Interviews will be transcribed verbatim in the source language by trained transcribers. Personal identifiers will be removed during transcription. Transcripts will then be translated into English for analysis. A random subset of transcripts will be independently reviewed by another study team member to check transcription accuracy and completeness, and that the original meaning is preserved during translation. Audio recordings will be permanently deleted after verified transcription and completion of quality assurance procedures.

Participants’ confidentiality will be strictly protected through de-identification and secure data storage practices. Quantitative data in REDCap and de-identified qualitative transcripts will be archived in a secure and access-controlled central office repository for 5 years following the date of final publication.

### Data analysis

The quantitative analysis of effectiveness will be based on intention-to-treat with pairwise comparisons of the three arms. Descriptive statistics such as the mean, standard deviation, median, and range will be presented for continuous variables. The intervention effects on outcomes will be assessed using mean differences for continuous outcomes and risk ratios (RRs) and absolute risk differences for binary variables. Data will be analysed using STATA/SE version 15 (StataCorp LLC, College Station, TX).

Efforts will be made to minimize missing data. Primary analyses will be based on complete case analysis. To evaluate the robustness of our findings, we will perform sensitivity analysis using inverse probability weighting.

For analysis of secondary outcomes at 7 and 18 weeks, participants in the SAATHI-I and SAATHI-IG arms will be combined and considered as a single intervention group since the participants in the latter arm will not have completed the monthly group meetings, which extend to 11 months.

We will also conduct subgroup analyses to examine differential effects of the intervention by sexual identity (gay men vs. transgender women).

The qualitative analysis of data from the formative research and process evaluation will be based on reflexive thematic analysis, grounded in a critical realist/contextualist ontology where interviewees’ experiences are understood as lived realities that are produced and exist within broader social contexts [72–74]. The transcribed material will be managed in QSR NVIVO 13. We will conduct a reflexive thematic analysis focusing on phenomenological descriptions and narrative constructions through the following steps: (1) The interviewers note their immediate impressions and responses after the interviews to increase reflexive awareness of interpersonal processes in the interview situation. (2) Researchers read the transcribed material and the interviewers’ notes to obtain a basic sense of the interviewees’ experiences and how they give meaning to the intervention and associated processes. (3) Meaning units will be coded and the codes merged into themes. The themes will then be reviewed by the research team and refined to ensure that they capture the range of perspectives represented in the data. A second researcher will independently audit the thematic structure. The final themes will be synthesized into written text.

### Monitoring

The SAATHI trial has established two independent oversight committees: a Data Safety Monitoring Board (DSMB) and Steering Committee. The members of both bodies are independent of the trial’s sponsor and funder and include academic and professional experts in relevant fields such as public health, violence prevention, mental health, and biostatistics.

The DSMB holds several key responsibilities, which are to protect participant safety, monitor study progress, review adverse events, and ensure data integrity. This board will determine whether the SAATHI trial is being conducted safely. The group will conduct safety reviews in closed sessions. Following these reviews, the DSMB will communicate its recommendations to the principal investigator and the Steering Committee. No interim analysis of the outcomes data is planned; a final analysis will occur after the last participant has completed follow-up.

Meanwhile, the Steering Committee is responsible for ensuring adherence to good clinical practice and monitoring ethical compliance. Another of its core duties is to review and provide feedback on key study documents. The Steering Committee will meet at least twice a year. Together, these two committees will provide coordinated oversight of the trial’s conduct, data quality, participant safety, and scientific integrity through joint meetings and exchange of reports.

### Ethics

Prior to the commencement of the study, ethical approval was obtained from the Nepal Health Research Council (NHRC) and the Regional Ethics Committee of Western Norway (REK). Peer advocates and field researchers were oriented about ethical considerations such as sensitive behaviour when approaching participants, creating a comfortable and friendly environment, and maintaining confidentiality and privacy. Field researchers will obtain written informed consent from all participants prior to study participation. Participants with no formal education will be provided with the option of witnessed written consent. Considering the vulnerable and marginalized position of LGBTIQA +, we will ensure that participation in the trial will not lead to anyone’s sexual identity being revealed against their own will. We will ask the participants at all follow-up assessments about any adverse experiences they have had in relation to their participation in the study in order to introduce counter-measures as soon as we can. The close collaboration with the community-led organizations is also likely to help us avoid any unintended negative effects. Any important protocol modifications will be submitted for approval to the NHRC and REK prior to implementation.

If the intervention is found to be effective in the intervention arms, the study team will work with the community-led organizations and trained peers to deliver intervention sessions to all the participants in the EUC. Additional funding will be sought to support the trained peer advocates in providing assistance to all eligible and interested LGBTIQA + individuals for a defined period.

The project will be managed according to the Montreal Statement on Research Integrity in Cross-Boundary Research Collaboration.

### Dissemination plan

The study findings will be disseminated among community-led organizations, healthcare professionals, program implementers, policy-makers, and other key stakeholders in Nepal via plain-language summaries, press releases, meetings, and policy briefs to inform advocacy, guidelines, and policy-making. Furthermore, the findings will be disseminated via scientific publications in high-impact international peer-reviewed journals and conference presentations for a global scientific audience.

## Discussion

This study will be the first study to adapt the PM + intervention to LGBTIQA + populations in a LMIC and to assess the feasibility and effectiveness of engaging peer counsellors in delivering enhanced SAATHI intervention to address mental health problems in this group. The evidence obtained from the trial is likely to also be highly relevant for LGBTIQA + in other settings where the exposure to violence is high. The SAATHI intervention will target both the causes and consequences of mental health problems among LGBTIQA +.

The use of peers as counsellors appears to be a promising, innovative, and cost-effective intervention to support LGBTIQA + individuals who are afraid to disclose about their identity. Studies on the involvement of peers in addressing mental health issues among LGBTIQA + have not been conducted in LMICs. The study findings might bridge the gap in mental health service providers for LGBTIQA +, not only in Nepal but in other LMICs too. The study will also aid in building the capacity of the community-led user organizations that will be involved during the preparatory and implementation phases of the study and the final dissemination of study findings. This will ensure that the interventions will be relevant for their needs and also minimize the risk of any unintentional harm. In addition, the intervention is expected to foster capacity building and empowerment within the LGBTIQA + community by training peer advocates who can continue to support community members beyond study period. We do not expect scalability to be a problem although resources in most LMICs are limited because this will be a low-cost intervention that can be implemented by the LGBTIQA + community itself.

We have identified and considered several potential barriers to the successful adaptation and implementation of the intervention. Addressing mental health disparities requires a focus on populations who are hard to reach within and outside formal health care systems. We anticipate that recruitment of transgender women may be more straightforward, whereas lesbians and gay men may be more difficult to identify and enrol. However, considering that we will recruit participants through user organizations, we assume that other groups than transgender women will also come forward.

There is a potential risk of contamination between study arms due to highly networked nature of the study population. Participants in the intervention arm may share information, skills, or coping strategies with peers who are enrolled in the control arm. We will attempt to minimize this risk through clear messaging during enrolment and by reinforcing the importance of not sharing intervention materials; however, some level of contamination remains possible in community-based trials. Participants in the control arm may also seek counselling or other psychosocial support services outside the study. Participants in the EUC arm will be interviewed after their final follow-up assessment and asked whether they became aware of any strategies used in the SAATHI intervention, how they received this information and from whom. These data will allow us to assess the extent and potential impact of contamination between study arms. Another risk is differential attrition. Participants in the control arm may feel they are not receiving direct therapeutic benefit and may therefore be more likely to drop out. We will document reasons for attrition where possible to help interpret results.

### Trial status

Recruitment started in May 2025. As of 29 December 2025, 1054 participants have been enrolled. Participant recruitment is expected to be completed by early January 2026, and final follow-up will be completed by January 2027.

## Supplementary Information

Supplementary Material 1.

Supplementary Material 2.

## Data Availability

Collaborators and qualified independent researchers may request access to the de-identified individual participant data and supporting documentation (e.g., codebooks and analysis plan) by submitting a reasonable written request to the principal investigator (PI). Contact information will be provided in the scientific publications associated with the study. To protect participants’ privacy, a de-identified dataset will be shared only after the publication of findings related to primary and secondary outcomes. The shared dataset will exclude identifying variables such as age, study district, and gender identity.

## Abbreviations

BDS: Blue Diamond Society
CBPR: Community-based participatory research
DSM-5: Diagnostic and Statistical Manual of Mental Disorders, Fifth Edition
DSMB: Data Safety Monitoring Board
EQUIP: Ensuring Quality in Psychosocial and Mental Health Care
ENACT: Enhancing Assessment of Common Therapeutic factors
EUC: Enhanced Usual Care
GHQ-12: General Health Questionnaire (12-item)
IPV: Intimate partner violence
LMICs: Low- and middle-income countries
LGBTIQA +: Lesbian, Gay, Bisexual, Transgender, Intersex, Queer, Asexual and other sexual and gender diverse identities
MSSS-5: Modified Social Support Survey (5-item version)
NHRC: Nepal Health Research Council
PCL-5: PTSD Checklist for DSM-5
PM +: Problem Management Plus
PSYCHLOPS: Psychological Outcomes Profile
PTSD: Post-traumatic stress disorder
RCT: Randomized controlled trial
REDCap: Research Electronic Data Capture
REK: Regional Ethics Committee (Western Norway)
RR: Risk ratio
SAATHI: Support, Advocacy, and Awareness for Trauma, Healing and Inclusion
SAATHI-I: SAATHI Individual Counselling
SAATHI-IG: SAATHI Individual Counselling plus Group Meetings
WHO: World Health Organization
WHODAS 2.0: WHO Disability Assessment Schedule 2.0
