# Supplementary material for: Effectiveness of peer counselling in addressing psychological distress and exposure to violence among sexual and gender minorities in Nepal: a study protocol for a randomized controlled trial
Source: Trials. 2026 Jun 16;27:545. doi: 10.1186/s13063-026-09843-9 (PMC13505074; doi:10.1186/s13063-026-09843-9)
Supplement: Supplementary file 2 [file 13063_2026_9843_MOESM2_ESM.docx]

**Supplementary file 2. Summary of SAATHI individual counselling**

| Session 1 | Introduction and talk about confidentiality by using basic helping skills. Introduction of the SAATHI program and PSYCHLOPS 1 assessment. Understanding adversity (discuss about violence and mental health including the concept of self-acceptance and self-compassion). Provide psychoeducation on violence and mental health. Discussing cyclical nature of violence. Use of case examples to clarify clients about the problems and to provide a scenario for more clarifications. Introduce “Managing Stress” via practicing slow breathing techniques and other relaxation techniques; also discuss about implementation intention. Demonstration of different forms of slow breathing techniques. Revising the session and remind about the next session. |
| --- | --- |
| Session 2 | General conversation and reminding about confidentiality and privacy. PSYCHLOPS 2 assessment including assessment of suicide. Tailored assisted and accompanied referral will be provided for client with suicidal thought and plan. Managing stress via different forms of stress management techniques like slow breathing, “*anulom-vilom”,* mindful meditation. Review the slow breathing documentation form provided in session 1 and hand over next blank slow breathing documentation form for updating the breathing exercises performed this week. Managing problems (Identifying solvable and unsolvable problem; start working on client’s main solvable problem by adopting problem solving steps and using basic helping skills). Work through client’s problem by using motivational interviewing approach (Open questions, affirmations, reflective listening and summary reflections). Revising the session and remind about the next session. |
| Session 3 | Reminding about confidentiality and privacy. PSYCHLOPS 2 assessment including assessment of suicide. Tailored assisted and accompanied referral will be provided for client with suicidal thought and plan. Review of managing problem strategy. Discuss if the strategy worked or not at solving the identified problem. If the strategy did not work, try to solve the problem with next identified solution. Introduce “get going, keep doing” strategy (activities aimed at breaking the cycle of inactivity) for behavioral activation. Breaking one larger task into small and manageable activities. Review and practice stress management activities particularly focusing on slow breathing strategies including mindfulness strategy. Revising the session and remind about the next session. |
| Session 4 | Reminding about confidentiality and privacy. PSYCHLOPS 2 assessment including assessment of suicide. Tailored assisted and accompanied referral will be provided for client with suicidal thought and plan. Review of managing problem. Review of “get going, keep doing”. Review the weekly calendar form filled up by the client. Introduce strengthening social support strategy, identifying formal and informal support available. Identify the support system to whom client can rely on during the difficult times and also conduct a role-play on how to approach the identified support system. Also provide practical information on available support system. Review and practice stress management activities particularly focusing on slow breathing strategies including mindful meditation. Revising the session and remind about the next session. |
| Session 5 | Reminding about confidentiality and privacy. PSYCHLOPS 2 assessment including assessment of suicide. Tailored assisted and accompanied referral will be provided for client with suicidal thought and plan. Review of managing problem. Review of “get going, keep doing”. Introducing self-empowerment. Safety behavior assessment by using safety behavior checklist. Discussion on barriers and facilitators of safety planning including its importance and also prepare a fictional safety plan. Review and practice slow breathing strategies. Revising the session and remind about the next session. |
| Session 6 | Reminding about confidentiality and privacy. Review previous sessions including the strategies discussed and how it has changed the lives of the client. Staying well (discuss about healthy habits) and imagining how to help others (an imaginary discussion on how the client will help someone facing similar problems by using a case example). Looking to the future (conversation on positive thoughts and client’s goal for the future). Review and practice slow breathing strategies. Ending the sessions and remind about the next follow up visit. |
